# Telemedicine minimum viable product for post-exposure prophylaxis to biological material in the COVID-19 pandemic

**DOI:** 10.47626/1679-4435-2023-1229

**Published:** 2024-11-14

**Authors:** Flávio Henrique de Holanda Lins, Tiago Pessoa Lima, Allan Rodrigo dos Santos Araujo, Suely Arruda Vidal, Maria Júlia Gonçalves de Mello

**Affiliations:** 1 Graduate Program (Stricto Sensu), Instituto de Medicina Integral Prof. Fernando Figueira, Recife, PE, Brazil; 2 Outpatient Clinic, Hospital Correia Picanço, Recife, PE, Brazil; 3 Department of Systems Analysis and Development, Instituto Federal de Pernambuco, Palmares, PE, Brazil; 4 Master’s Program in Software Engineering, Centro de Estudos e Sistemas Avançados do Recife, Recife, PE, Brazil

**Keywords:** accidents, COVID-19, HIV, post-exposure prophylaxis, telemedicine, acidentes, covid-19, HIV, profilaxia pós-exposição, telemedicina

## Abstract

**Introduction:**

The COVID-19 pandemic brought new challenges for health services. The follow-up of cases of exposure to biological material is regulated by protocols of the Ministry of Health in Brazil. Telemedicine can be useful in maintaining appointments and reducing patient crowding.

**Objectives:**

To describe the development and implementation of telemedicine as a minimum viable product to care for victims of exposure to biological material in the context of the COVID-19 pandemic, in a reference service in northeastern Brazil.

**Methods:**

Descriptive study of the development of a minimum viable telemedicine product, seeking testimony from those involved and reviewing the script and tools used.

**Results:**

The telemedicine system was developed as a minimum viable product and worked asynchronously, being developed by integrating the WhatsApp Business, YouTube and Google Forms platforms and associating them with Excel and Word using database and direct mail tools, in addition to electronic equipment, cell phone, laptop, printer and scanner, and provided the follow-up of 742 cases of exposure to biological material from July 2020 to July 2021.

**Conclusions:**

The integration of tools already available on the internet allows a professional outside the field of information technology to develop a minimum viable telemedicine product, aiming to maintain health care and avoid the crowding of patients in outpatient clinics. We suggest the development of a robust tool that integrates several routines for monitoring cases of exposure to biological material.

## Abstract (pt)

**Introdução:**

A pandemia da covid-19 trouxe novos desafios para os serviços de saúde. O seguimento dos casos de exposição a material biológico no Brasil é regulado por protocolos do Ministério da Saúde. A telemedicina pode ser útil na manutenção dos atendimentos evitando a aglomeração de pacientes.

**Objetivos:**

Descrever o desenvolvimento e implantação de telemedicina como produto mínimo viável para atendimento de vítimas de exposição a material biológico no contexto da pandemia da covid-19, em um serviço de referência no nordeste do Brasil.

**Métodos:**

Estudo descritivo do desenvolvimento de um produto mínimo viável de telemedicina, buscando depoimento dos envolvidos e revisando o roteiro e ferramentas utilizadas.

**Resultados:**

O sistema de telemedicina foi desenvolvido como um produto mínimo viável e funcionou de forma assíncrona, integrando as plataformas WhatsApp Business, YouTube e Google Forms. Além disso, o sistema foi associado ao Excel e ao Word utilizando ferramentas de bancos de dados e mala direta, bem como equipamentos eletrônicos, telefone celular, *notebook*, impressora e *scanner*. O sistema proporcionou o acompanhamento de 742 casos de exposição a material biológico no período entre julho de 2020 e julho de 2021.

**Conclusões:**

A integração de ferramentas já disponíveis na internet possibilita que um profissional fora da área de tecnologia da informação desenvolva um produto mínimo viável de telemedicina. Isso é crucial para manter a atenção à saúde e evitar a aglomeração de pacientes nos ambulatórios. Sugerimos o desenvolvimento de uma ferramenta robusta que integre várias rotinas do acompanhamento dos casos de exposição a material biológico.

## INTRODUCTION

Following the declaration by the World Health Organization (WHO), the COVID-19 pandemic was officially recognized in March 2020.^1^ In Brazil, health authorities had to take measures, such as suspending elective consultations, to reduce the spread of COVID-19 and its serious impact on the population’s health.^2,3^ In the state of Pernambuco (PE), one of the services affected by this measure was the outpatient clinic of Reference Service for Accidents with Exposure to Biological Material (SREMB) at Hospital Correia Picanço (HCP), resulting in a significant backlog of cases requiring follow-up. This situation became even more critical when more than 250 cases of the “carnival needle spiking” phenomenon were reported in 2020,^4^ preventing the provision of care within the planned timeframe.

Follow-up of these accidents follows a well-defined standardization by the Brazilian Ministry of Health (MS) through the Clinical Protocol and Therapeutic Guidelines (PCDT) for postexposure prophylaxis (PEP) to biological material. The primary role of the physician in the outpatient clinic is to provide guidance, clarify doubts, and request and provide the results of follow-up tests. Reliable information usually reduces patient anxiety and builds confidence for complete follow-up. Reports of clinical complaints during follow-up are very rare, so physical examinations are almost never required.^5^ These characteristics facilitate the use of telemedicine resources for the follow-up of such cases.

Wosik et al.^6^ list the following types of telemedicine: e-consultation, which is asynchronous physician-to-physician communication based on review of patient records (both inpatient and outpatient); remote patient monitoring, which involves collection of patient data outside the traditional health care setting via connected devices or patient-reported outcomes (synchronous or asynchronous); patient-initiated messaging, which involves synchronous chat with automated or live agents; or asynchronous communication.

In his book *The Lean Startup*,^7^ Ries discusses the fundamentals of improving the success of startups, which often operate in a highly uncertain environment. One of the proposed methodologies is the minimum viable product (MVP), a release that allows for a complete cycle of build-measure-learn with minimal effort and development time, thereby enabling agility in development while reducing overall costs.

The aim of this study was to describe the development and implementation of telemedicine as a MVP for the care of victims of exposure to biological material (EBM) at SREMB-HCP in the context of the COVID-19 pandemic.

## METHODS

This descriptive study was conducted to develop a telemedicine MVP for the outpatient follow-up of EBM cases, as a contingency to the suspension of in-person appointments at HCP in Recife, capital city of PE. The development of the intervention took place between April and July 2020, and its implementation began in the second half of July 2020.

HCP is a unit of the Brazilian National Health System (SUS) in PE. HCP is specialized in infectious diseases, especially AIDS/HIV and meningitis. Since 2004, a specialized outpatient clinic has been established for the follow-up of accidents involving EBM. The HCP emergency care service (ECS) receives cases of occupational EBM (OEBM), which is a notifiable condition, community EBM (CEBM), and high-risk sexual exposure (HRSE) through walk-in visits. During the consultation, the type of exposure is characterized, tests are performed, and antiretroviral (ARV) medications are dispensed if indicated. The SREMB HCP is responsible for 85% of PEP prescriptions for EBM in PE.^8^

Outpatient follow-up is based on the protocol defined by the MS,^9^ and reporting to the Brazilian Information System for Notifiable Diseases (SINAN)^10^ is done by the Hospital Epidemiological Surveillance (VEH). The initial reception and care of walk-in cases in the ECS was maintained during the COVID-19 pandemic, while the focus of telemedicine was on outpatient follow-up.

Script and tools used were reviewed, including WhatsApp Business, YouTube, Google Forms, and forms generated by Word mail merge based on data recorded in Excel spreadsheets. Before starting the implementation of telemedicine at SREMB-HCP, approval was obtained from the hospital administration.

Research project was approved by the Research Ethics Committee of Instituto de Medicina Integral Prof. Fernando Figueira (IMIP) (Certificate of Ethics Review 53388421.8.0000.5201 and approval opinion no. 5.549.579).

## RESULTS

The implemented telemedicine system has been designed as an MVP^7,11^ and operated asynchronously, integrating platforms such as WhatsApp Business, YouTube, and Google Forms as well as linking Excel spreadsheets with Word text editor by using database and mail merge tools. In addition, electronic devices such as mobile phones, laptops, printers, and scanners were used. The system was designed by the physician in charge of SREMB-HCP.

After initial consultation at the ECS, the patient was instructed to send a message to the WhatsApp number to start the outpatient follow-up. Through the same channel, the patient received general instructions, links to YouTube videos, and a link to fill out the medical form via Google Forms, either through automated messages generated by WhatsApp Business (Chart 1) or through direct intervention by the professional with personalized messages.

**Chart 1:** Summary of the main automated or preformatted text messages on WhatsApp Business for the telemedicine system at Hospital Correia Picanço (HCP)

Initial contact	Medical form	Tests - requests and results
Automated message Hospital Correia Picanço (HCP) Outpatient clinic Dr. Flávio No in-person consultations, only telemedicine available. The first reassessment is conducted between 30 to 45 days after the event. If taking the cocktail, continue until completing 28 days. Sexual activity only with a condom and no blood donation until cleared from the accident/exposure. Subscribe to our YouTube channel and watch informational videos: https://www.youtube.com/playlist?list=PLxy4frfUx0kDSyRRvURp0KpaeBzFZ9o3G If this is your first contact, please provide (otherwise, state the reason for contact): 1. Full name 2. Date of attendance at ECS of HCP 3. Type: a) occupational accident b) sexual exposure c) community accident or assault Save this number on your phone I respond on Tuesday and Thursday mornings (sometimes outside these hours)	Occupational accident Consultations still unavailable. For telemedicine, please fill out the medical form at the link below: https://forms.gle/xthyt6YqhGszD82E8 The form is simple to complete. If you need help, there is a link to an explanatory video: https://youtu.be/_t3hDuZDr5o (also available in the first part of the form). After submission, wait up to 3 business days for a response on the next steps. Non-occupational accident Consultations still unavailable For telemedicine, please fill out the form at the link below: https://forms.gle/Msmf6E8XXh9D9HQ7A The form is simple to complete. If you need help, there is a link to an explanatory video: https://youtu.be/huhqxukLqZ4 (also available in the first part of the form) After submission, wait up to 3 business days for a response on the next steps. Sexual exposure Consultations still unavailable For telemedicine, please fill out the medical form at the link below: https://forms.gle/9nrzEA6x4vQKh3o89 The form is simple to complete. If you need help, there is a link to an explanatory video: https://youtu.be/ZKtBTYIiYpQ (also available in the first part of the form) After submission, wait up to 3 business days for a response on the next steps.	(Send photo of the test request followed by text) Go directly to the laboratory reception at Hospital Correia Picanço on the date of the request (see photo) (between 8:30 am and 10 am). Fasting is not required. When the result is ready, I will be notified and will contact you via WhatsApp with further instructions. Rua Padre Roma, 149 Tamarineira Recife PE. First-come, first-served basis. For rescheduling, call: 81 3184 3964 from 9 am to 12 pm. Results via WhatsApp in 30 to 45 days after collection. No need to print the guide. Your first reassessment tests conducted on: dd/mm/yy are all normal. Paper results will be attached to the test guide at the laboratory reception on the day of the next collection. Next step is to redo the tests 90 days after exposure. Request will be sent soon. Note the tests cannot be collected before the date noted on the request. The recommendation to avoid blood donation and use condoms continues! Your 3-month follow-up tests conducted on: dd/mm/yy are all normal. Paper results will be attached to the test guide at the laboratory reception on the day of the next collection. With this, I can clear you for HIV. Only a hepatitis test remains, to be conducted 6 months after exposure. Request will be sent soon. Note the tests cannot be collected before the date noted on the request. The recommendation to avoid blood donation and use condoms continues! (Send photo of test results followed by text): Considering the time between the exposure/accident event and these test results, I can clear you. Congratulations. Normal life. Save this number to receive messages on the broadcast list. Soon, you will receive a survey about this type of service.

Chart 1 shows a summary of the main automated or preformatted text messages on WhatsApp Business to streamline the service.

YouTube videos were created to replicate the routine dialog typically established with patients. For this purpose, a YouTube channel (Mat Bio) was created, and the topics were divided into nine videos, ranging in length from 50 seconds to 17.5 minutes (Chart 2). A total of 24 hours of work was invested, including 8 hours of learning about YouTube Creators,^12^ 8 hours of learning how to use the necessary tools (e.g., Canva^13^ for layout design and ShotCut^14^ for video editing), and 8 hours of recording and editing the videos.

**Chart 2:** Index of videos on the Mat Bio YouTube channel used in the telemedicine system implemented at Hospital Correia Picanço (HCP) in response to COVID-19 pandemic

Order	Title	Duration (min)
1	Introduction to Mat Bio channel	00:50
2	Types of services at reference center for exposure to biological material	17:20
3	Why are exposures to biological material considered a medical emergency?	04:35
4	Types of biological material	03:48
5	Transmission risks in accidents involving exposure to biological material	11:06
6	Sexual exposure: transmission risks	05:55
7	Who is the source patient?	04:43
8	Outpatient follow-up for patients exposed to biological material	04:37
9	Needle spiking incidents during carnival in Recife and Olinda	08:57

Chart 2 shows the titles of the videos on the Mat Bio YouTube channel, along with their respective durations in minutes.

Specific forms have been created in Google Forms to capture all of the data that is typically collected during in-person consultations and provide a basis for appropriate follow-up. The Google Forms clinical record templates are available at the following links: occupational accident (https://forms.gle/hgryTfoA8aXqvTaC9); community accident (https://forms.gle/2NVxustfsCLns2of9); and sexual exposure (https://forms.gle/ehTGZZa1URKwD2TL7). Data collected from the electronic forms were transferred to an Excel spreadsheet, where additional data reported via WhatsApp Business messages, as well as follow-up test dates and results, were added. Patients then received follow-up test reminders (generated by Word mail merge based on Excel data) and results of the tests performed, along with instructions for next steps until discharge.

A flowchart was created that included scheduling with the HCP laboratory. The physician was responsible for sending a photo of the follow-up test request with the scheduled date and time to the patient’s WhatsApp. The request form was then printed and delivered to the lab receptionist, where it was placed in a folder. On the scheduled date, the patient would go to the lab, where the photo of the request on the patient’s WhatsApp was compared to the printed request form.

Since the laboratory did not have a web system for delivering results, the test results were sent to the SREMB HCP physician, who then delivered the results to the patients via WhatsApp Business. Since almost all follow-up tests for EBM are typically negative, there was no difficulty in delivering the results using this method. The printed test results were later provided at the lab reception desk when patients returned for the next phase of follow-up testing.

**Figure 1:**
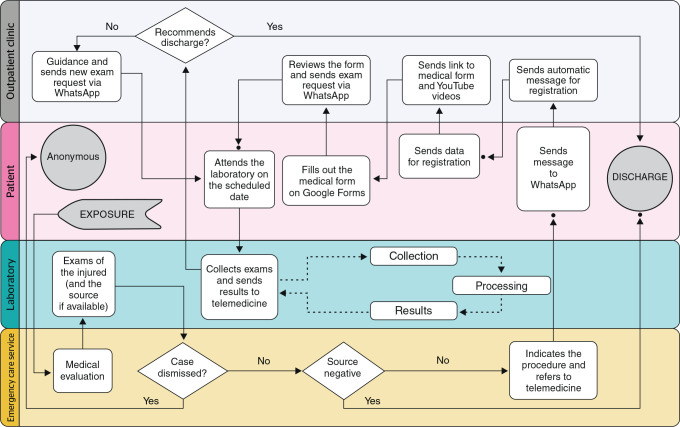
Workflow diagram of the telemedicine system implemented at Hospital Correia Picanço (HCP) in response to COVID-19 pandemic.

At case closure upon discharge, results were sent as photos via WhatsApp Business. In special situations requiring personalized care, patients were called in for face-to-face consultations, which were rare. Figure 1 shows the workflow diagram of the telemedicine service.

Forms used for data collection, test requests, and automated and standardized responses on WhatsApp Business were refined during the period of telemedicine availability, based on feedback from both patients and professionals involved. After the telemedicine system was discontinued, data were printed in the standardized form formats used by the service, with the support of Word mail merge function, and sent to HCP archive.

Between July 2020 and January 2022, the SREMB-HCP telemedicine MVP facilitated the follow-up of 742 EBM cases. During this period, the Mat Bio YouTube channel, which hosted the videos used for telemedicine, had 112 subscribers, 3,683 views, and 9,294 minutes of viewing time. In addition, WhatsApp Business recorded 23,344 messages sent and 20,974 messages received.

The master’s thesis that led to this article included two other study lines that evaluated the impact of the intervention on patient care and satisfaction, with satisfactory results.

## DISCUSSION

Sharing experiences aimed at solving problems that are likely to be common to many other health care services is important. Even if full replication is not possible, the visibility of the project can inspire and facilitate the search for specific solutions in other cases.

The telemedicine system implemented at HCP made it possible to continue providing care to a population that would have been completely underserved during an event like the COVID-19 pandemic. The professional responsible for the service had extensive knowledge of the entire routine, and the methodology recommended for startups in developing an MVP was critical to creating a simple and cost-effective solution.^7,11^

Managing EBM cases is straightforward, as it is systematized by the MS PCDT.^5^ However, it is often quite bureaucratic for the teams, especially in emergency care. Typically, the same information needs to be recorded in the treatment report, the test request form, the ARV request form, and the SINAN epidemiological notification form.^10^

These processes interfere with the decentralization of care, as occasional care providers often lack the necessary expertise or would spend too much time consulting manuals to perform the entire routine. As a result, patients travel long distances to this reference center for follow-up care, contributing to high dropout rates and increased costs.

In the synchronous model, as used in telepsychiatry, previously face-to-face consultations are conducted via video or voice. This model is generally limited by the difficulty of conducting physical examinations. The schedule of the professional performing the consultation must be coordinated with patient availability, and each professional can only see one patient at a time.

An asynchronous product provides patients the freedom to access services at the most convenient time for them. Another advantage is that automation and artificial intelligence resources allow a professional to manage the care of multiple patients simultaneously, optimizing the schedule of available human resources.^15^ The standardized PEP model for EBM, with minimal need for physical examination, is well suited to the asynchronous telemedicine model.

Hill et al.^12^ emphasize that recognizing and learning from often untold stories will lead to more efficient, effective, and sustainable mHealth (a term used for remote health care services) efforts. They invite researchers to document and share their own challenges and strategies. Among the principles adopted, they highlight the acceleration of an MVP to be tested and improved in stages.

The number of cases followed during the period was within the usual demand for the service. The high number of messages on WhatsApp Business is explained by the fact that each message sent is counted individually, and it is common to split a single topic into multiple messages. For example: “good morning,” “my name is so-and-so,” “I had an occupational accident,” and “it was on 09/05/2020.” In this case, the system counts four messages, whereas “good morning, my name is so-and-so, I had a work accident on 09/05/2020” is counted as only one message. The discrepancy between the low number of subscribers and the high number of channel views suggests patients may be prioritizing their privacy.

The proposed product has some limitations that need to be considered. One of these is digital exclusion, which may affect a proportion of EBM victims who do not have access to devices (e.g., mobile phones, computers, and the Internet) or who have difficulty using the available technologies.^16^ During the critical phase of the pandemic, when in-person consultations were suspended, the only option was to rely on the support of friends and family to use telemedicine. However, with the return of face-to-face services after this phase, it is expected that these users will be able to receive care again.

Another major limitation is data security. At the onset of the pandemic, many countries did not have clear regulations for telemedicine, leading to hastily created standards to enable the continuation of care.^17^ While these regulations were necessary during the critical phases of the pandemic, there are still doubts about their future validity. Sensible, balanced regulations are needed to ensure the protection of patients and their data without hindering the use of telemedicine.^18^

The return of face-to-face care at the HCP, along with discussions about data security, led to the discontinuation of the MVP presented here. However, the experience was valuable in suggesting the creation of a formal app by the state and/or federal government’s telemedicine department. This app should comply with the Brazilian General Data Protection Act and optimize the entire care process, from initial emergency care to outpatient and laboratory follow-up.

To develop a robust application for the follow-up of EBM cases, it is necessary to have a multidisciplinary team that can contribute to different areas. In addition to health care workers working in the field, it would be essential to include a) design professionals: they should evaluate all user interface elements to reduce usability issues and create a positive user experience; b) software engineering professionals: to evaluate the development proposal using coding strategies such as low-code or programming languages to ensure the application is robust, secure, and scalable; c) data scientists: they should understand user behavior and the information collected to enable predictions through the use of artificial intelligence and/or automation. These professionals can help develop diagnostic algorithms, identify risk factors, and continuously improve the application.

By working together, this team can develop an application that meets the needs of both users and healthcare professionals and contribute to the improvement of EBM diagnosis and treatment. The main goals would be: assist professionals in the initial care by suggesting the routines to be followed; streamline data collection, avoiding duplication, and allowing patients to enter part of their medical history directly into the app; improve the quality of data recording for epidemiological studies with an interface to SINAN.^10^

In addition to the previous objectives, it is important to: streamline the dispensing of ARVs^19^ with an interface to the pharmacy; systematize the stages of outpatient follow-up using automation and/or artificial intelligence to optimize care capacity; optimize the distribution of patients across health units based on their location and residence; implement referral and counter-referral actions to decentralize care; promote quick and easy training for professionals based on standardized procedures and routines using automation and/or artificial intelligence; organize patient data with confidentiality and ease of access for responsible professionals; reduce follow-up abandonment with tools for appointment and test collection date alerts; lower SUS costs by avoiding duplicate exams and procedures, reducing office supply usage, and minimizing physical storage needs.

**Figure 2:**
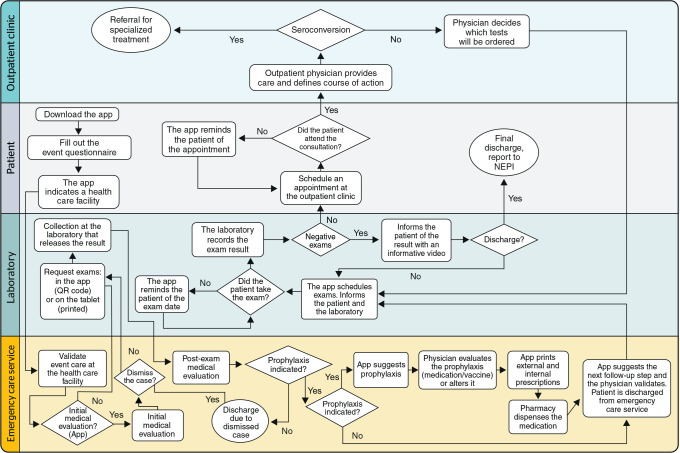
Suggested flowchart for developing an app for handling cases of exposure to biological material (EBM). NEPI = Núcleo de Epidemiologia.

Figure 2 shows a proposed flowchart for the development of an application to manage EBM cases, which could be piloted in a reference service such as HCP. The implementation of this app could be gradual, gradually fulfilling all the objectives, and then expanded geographically to the health regions of PE, until it is incorporated as a support tool for these programs at the national level within the SUS.

## CONCLUSIONS

The integration of readily available Internet tools allowed the development of a telemedicine MVP aimed at maintaining health care attention, avoiding patient crowding in clinics, and saving time and travel costs for patients.

The telemedicine MVP proved to be sufficient to maintain PEP follow-up for EMB and can serve as a basis for developing a robust tool that integrates different routines for follow-up of these cases. Moreover, it can be systematically implemented with a wide scope of action for SUS.
